# miR-644-5p carried by bone mesenchymal stem cell-derived exosomes targets regulation of p53 to inhibit ovarian granulosa cell apoptosis

**DOI:** 10.1186/s13287-019-1442-3

**Published:** 2019-11-29

**Authors:** Bo Sun, Yujia Ma, Fang Wang, Linli Hu, Yingpu Sun

**Affiliations:** 1 Center for Reproductive Medicine, The First Affiliated Hospital of Zhengzhou University, No.1, Jianshe East Road, Zhengzhou, 450052 Henan Province China; 2 Henan Key Laboratory of Reproduction and Genetics, The First Affiliated Hospital of Zhengzhou University, No.1, Jianshe East Road, Zhengzhou, 450052 Henan Province China; 3 Henan Provincial Obstetrical and Gynecological Diseases (Reproductive Medicine) Clinical Research Center, The First Affiliated Hospital of Zhengzhou University, No.1, Jianshe East Road, Zhengzhou, 450052 Henan Province China; 4 Henan Engineering Laboratory of Preimplantation Genetic Diagnosis and Screening, The First Affiliated Hospital of Zhengzhou University, No.1, Jianshe East Road, Zhengzhou, 450052 Henan Province China

**Keywords:** POF, miR-644-5p, Exosome, BMSC, p53

## Abstract

**Background:**

This article aims to reveal the therapeutic effects and potential mechanisms of bone mesenchymal stem cell (BMSC)-derived exosomes on premature ovarian failure (POF).

**Methods:**

Exosomes were collected from BMSCs and were used to treat cisplatin-induced POF mouse models. Pathological changes of ovarian tissue were detected by using HE staining and by Western blot that detected the expression of apoptosis-related proteins. In cisplatin-induced primary granulosa cell injury, exosomes were co-cultured with the granulosa cells. The apoptosis or viability of granulosa cells was analyzed by flow cytometry or MTT, respectively. In Target scan and microT-CDS databases, an intersection of miRNAs targeting to p53 was found. The expressions of miRNAs in BMSC-derived exosomes were detected by qRT-PCR. Besides, miR-664-5p targeted to p53 of cells was verified by dual-luciferase reporter assay.

**Results:**

BMSC-derived exosomes improved the follicular morphology of POF mice and inhibited the expression of apoptosis-related protein. By co-culture of exosomes and primary granulosa cells, BMSC-derived exosomes repressed cisplatin-induced granulosa cells apoptosis and increased cells viability, while these effects were abrogated after the exosome-containing RNA was degraded by RNase. By Target scan, microT-CDS and qRT-PCR, miR-664-5p was regarded as the dominated RNA in BMSC-derived exosomes. By dual-luciferase reporter assay, miR-664-5p negatively regulated p53 luciferase activity. After shRNA interfering miR-664-5p of BMSC, BMSC-derived exosomes exerted no protective effect on cisplatin-induced granulosa cell apoptosis.

**Conclusion:**

Our results indicated that miR-644-5p carried by BMSC-derived exosomes inhibited the apoptosis of ovarian granulosa cell by targeting p53 of cells, suggesting that miR-644-5p had the potential to treat POF and restore ovarian function.

## Introduction

Premature ovarian failure (POF) is caused by follicular depletion or dysfunction, and the clinical manifestations are amenorrhea, infertility, hot flashes and sweating, and decreased libido. According to statistics, the incidence of POF is about 0.1% in women aged 15–29 and 1% in women aged 30–39, which ultimately leads to female infertility [1]. Granulosa cells are a layer of parietal cells wrapped around the surface of follicles that support the formation and development of follicles and have the ability to secrete glandular hormones to maintain ovarian function [2]. Apoptosis of granulosa cells leads to follicular atresia, resulting in a decrease in the number of follicles [3]. Therefore, the inhibition of granulosa cells apoptosis is essential for improving POF.

Bone mesenchymal stem cells (BMSCs) are a subpopulation of cells with diverse differentiation potential and are a research hotspot in the field of stem cell therapy. Studies have shown that BMSCs can effectively restore the structure and function of damaged ovarian tissue in rats and inhibit the apoptosis of granulosa cells [3]. Other studies have found that BMSCs can slow the apoptosis of rat granulosa cells induced by cisplatin [4]. However, the mechanism by which BMSCs work remains unclear. Exosomes, membrane vesicles, have been reported to be secreted by BMSCs and mediate the therapeutic function of BMSCs through cell-to-cell communication and signal transduction [5, 6] in various diseases. For example, BMSC-derived exosomes have been reported to be effective in restoring neural function in stroke models of rats [7]. In addition, studies have indicated that BMSC-derived exosomes can promote the recovery of cognitive function in mice [8].

Recently, evidences show that the microRNAs (miRNAs) carried by exosomes mediated the signal transduction between cells and significantly affect the biological function of donor cells to the receipt cells in diseases [9, 10]. It has been reported that miR-223 carried by BMSC-derived exosomes can improve experimental autoimmune hepatitis [11]. Studies have shown that BMSC-derived miRNA-containing exosomes can increase the survival of retinal ganglion cells [12]. Recent studies have reported that microRNA-126-3p carried by BMSC-derived exosomes is effective in inhibiting the development of pancreatic cancer [13]. Another study finds that miR-10a carried by amniotic fluid stem cell-derived exosomes can inhibit the apoptosis of ovarian granulosa cells [14]. However, the miRNAs in BMSC-derived exosomes that affect the growth of granulosa cells are largely unknown. In this study, miR-664-5p was considered to be the major RNA in BMSC-derived exosomes by Target scan, microT-CDS, and qRT-PCR.

In this research, we found that miR-644-5p was dramatically overexpressed in BMSC-derived exosomes and affected the function of ovarian granulosa cells by targeting p53 of cells.

## Materials and methods

### BMSC culture and transfection

The isolation of BMSCs was based on methods established in previous studies [15]. BMSCs were resuspended in DMEM medium containing 10% FBS, and the cells were evenly distributed in the culture dish by shaking gently, and then statically cultured at 37 °C, 5% CO_2_.

To observe the effect of miR-664-5p carried by BMSC-derived exosomes on granulosa cells, we transfected shRNA-miR-664-5p into BMSCs and isolated the exosomes from BMSCs for the subsequent experiments.

### BMSC identification

BMSCs were identified by cell differentiation potential and surface markers. The specific method was to add 10% FBS, 5 μg/mL insulin, 0.1 μM dexamethasone, 0.2 mM vitamin C, and 10 mM β-glycerophosphate to IMDM to obtain an osteogenic induction medium. During the process of osteogenic differentiation, calcium ions could precipitate and form calcium nodules, and the potential of osteogenic differentiation of cells could be discriminated by the color reaction of alizarin red with calcium and the production of deep red-colored compounds. The specific method was to add 10% FBS, 10 μg/mL insulin, 1 μM dexamethasone, 0.5 mM IBMX, and 0.1 mM indomethacin to IMDM to obtain an adipogenic induction medium. The solution was changed once every 3 days and differentiated for 12 days. During the process of adipogenic differentiation, oil droplets appeared in the cytoplasm of cells. The oil droplets were subjected to target staining by oil red O staining to discriminate the potential of adipogenic differentiation of cells. In addition, flow cytometry was used to detect the expression of BMSC surface markers CD34 and CD90.

### Isolation and identification of BMSC-derived exosomes

The above BMSCs isolated were cultured in DMEM supplemented with 10% FBS and expanded to the third passage to extract exosomes. Specifically, the supernatant was collected and centrifuged at 3000*g* for 15 min, and the supernatant was mixed with the ExoQuick exosome precipitation solution. After centrifugation of the ExoQuick mixture at 1500*g* for 30 min, the supernatant was gently aspirated. Exosomes were isolated from the remaining ExoQuick solution by centrifugation at 1500*g* for 5 min. Finally, the exosomes were resuspended in PBS. The isolated BMSC-derived exosomes were identified based on the morphological characteristics observed by TEM and the detection of exosomes surface markers by Western blot.

### Establishment of cisplatin-induced POF mouse model

Fifteen C57BL/6 mice were from the Experimental Animal Center of Henan Province and divided into three groups after 6–7 weeks of feeding. The control group (*n* = 5) was intraperitoneally injected with normal saline. POF group (module group, *n* = 5) was intraperitoneally injected with 5 mg/kg cisplatin, and 100 μL PBS was injected into the tail vein on the 1st, 5th, and 10th day after modeling. The POF + BMSC exosome group (treatment group, *n* = 5) was intraperitoneally injected with 5 mg/kg cisplatin, and then exosomes (125 μg dissolved in 100 μL PBS) were injected into the tail vein on the 1st, 5th, and 10th day after modeling. Fifteen days later, the mice were sacrificed and the blood from the eyeball and ovarian tissues were taken for the subsequent experiments. Our research was approved by the Medical Ethics Committee of the First Affiliated Hospital of Zhengzhou University.

### Ovarian histology analysis

The collected ovarian tissue was fixed overnight in a Bouin solution (containing 5% acetic acid, 9% formaldehyde, and 0.9% picric acid), embedded in paraffin and serially sectioned ( 4 μM). After HE staining, the morphological structure of the ovary was observed under an optical microscope.

### Immunohistochemistry

Mouse ovarian tissue was fixed, embedded in paraffin, and cut into 4 μm thick. After deparaffinization and rehydration, the sections were taken, then incubated with the antibody, and then immunohistochemically tested according to the kit supplier’s instructions.

### Isolation, culture, and different treatment of granulosa cells

The mice were euthanized to collect the ovarian tissue, and a 30-gauge needle was used to pierce the follicles and granulosa cells were obtained under a stereoscopic microscope. Two microliters of MEM-α medium (containing 3.7 mg/mL NaHCO_3_, 10% (v/v) FBS, 100 IU/mL penicillin, and 100 mg/mL streptomycin) was added to granulosa cells and placed them in an 37 °C incubator with 5% CO_2_. Two microliters of non-adherent cells were aspirated every 3 days and then 2 mL of fresh medium was added to continue the culture.

In order to clarify the function of exosomes to protect granulosa cells from cisplatin injury, 10 μg/mL cisplatin, granulosa cells, and 10 μg/mL exosomes were co-cultured for 48 h to detect granulosa cell viability or cell apoptosis, respectively.

### Flow cytometry assay

Cisplatin-induced granulosa cell apoptosis was determined by flow cytometry using a FITC Annexin V/PI Apoptosis Detection Kit according to the manufacturer’s protocol. Briefly, cells were seeded in 6-well plates at a density of 4 × 10^5^. After 48 h, the cells were washed two to three times with cold PBS and stained with FITC Annexin V and propidium iodide (PI) for 15 min at room temperature using the Annexin V-FITC Apoptosis Detection Kit. The stained cells were analyzed using flow cytometry.

### Cell viability assay

After the cells were seeded in 96-well plate at a density of 4 × 10^4^ for 48 h, 5 mg/mL of MTT was added to the mouse ovarian granulosa cells and incubated at 37 °C for 4 h. Subsequently, the medium was discarded, and then incubation was continued for 6 min at room temperature after adding DMSO. Absorbance was measured at 570 nm with a μQuant microplate reader (BioTek Instruments, Winooski, VT).

### RNase treatment of BMSC-derived exosomes

After exosomes were treated with 100 μg/mL RNase A for 1 h at 37 °C, 400 U/mL RNase inhibitor was added to terminate the reaction. Finally, the exosomes were washed by centrifugation.

### Western blot

Ovarian granulosa cells were lysed by placing them in a whole lysis buffer containing a protease inhibitor cocktail (Sigma). Protein concentration was measured using the Pierce BCA Protein Assay (Thermo). SDS-PAGE gel electrophoresis was used to separate proteins and transfer to PVDF membranes at 4 °C. The membrane was then blocked with 5% skim milk for 1 h and then the membrane was incubated with primary antibody overnight at 4 °C. Subsequently, the membrane was further incubated with horseradish peroxidase conjugated secondary antibody for 1 h at room temperature, and finally, the band intensity was quantified using Image Lab software (Bio-Rad Laboratories).

### Enzyme-linked immunosorbent assay (ELISA)

The concentration of E2 in the mouse eye serum samples taken above was determined according to the ELISA kit supplier’s instructions (R&D Quantikine, R&D Systems Inc., Minneapolis, MN, USA).

### Quantitative real-time PCR (qRT-PCR)

Twenty-one overlapping miRNAs were screened by Target scan and microT-CDS, and miRNAs with the most significant differences in expression between BMSCs and 3 T3 exosomes were identified by qRT-PCR. Total RNA from exosomes was extracted using Trizol reagent. Total RNA was reverse transcribed into cDNA using the PrimeScriptTM RT kit. Real-time PCR was performed using SYBR Premix Ex Taq II (TaKaRa, Shiga, Japan) and Applied Biosystems 7500 Fast Real Time PCR System. The 2^-ΔΔCT^ formula was performed to calculate the relative expression levels.

### Dual-luciferase reporter (DLR) assay

Luciferase reporter plasmid containing the trp53 3′UTR was constructed and the trp53 3′UTR containing the mmu-miR-664-5p binding site mutation was cloned into the same reporter plasmid. The miR-664-5p mimic (miR-664-5p overexpression sequence) was subjected to lipofection with Lipofectamine 2000 (Invitrogen) and the above recombinant vectors were transfected into mouse ovarian granulosa cells. Subsequently, luciferase activity was measured using a Dual-Glo Luciferase kit (Promega) 48 h after transfection.

### Statistical analysis

SPSS 13.0 was used for statistical analysis, and the data were presented as mean ± standard deviation (SD). When two groups were compared, the Student’s *t* test was used to analyze the differences between the groups. When multiple groups were compared, the differences among the groups were assessed by using one-way ANOVA. *P* < 0.05 was considered to indicate a statistically significant difference. All experiments were performed three times.

## Results

### Extraction and identification of BMSCs and its exosomes

**Fig. 1:**
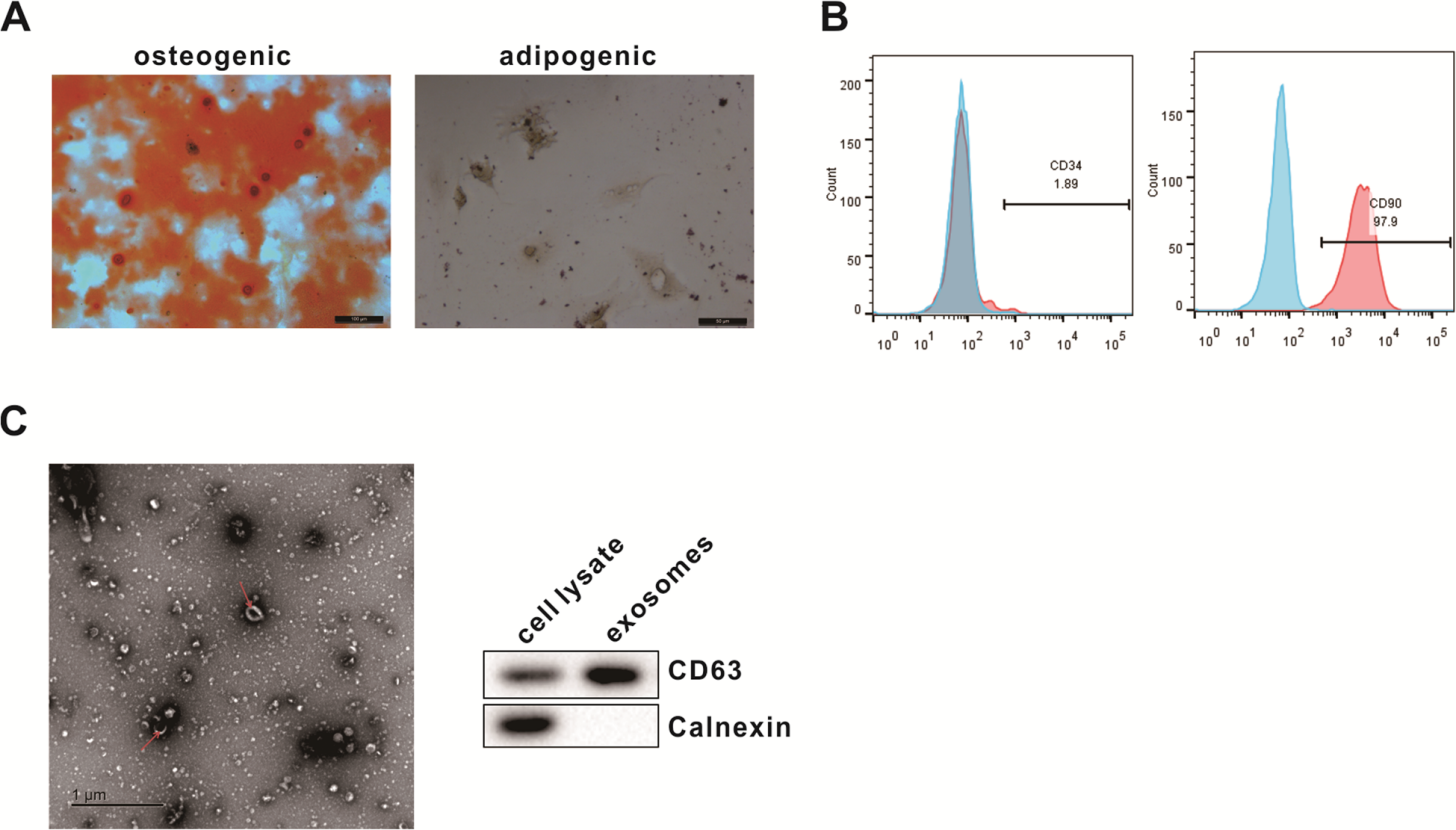
Identification of mouse BMSC-derived exosomes. **a** After the osteogenic and adipogenic induction medium was added into the cells, the morphology of the cells was observed under a microscope (scale bar, 100 μm and 50 μm). **b** Flow cytometry was used to detect the expression of BMSC surface markers. **c** Transmission electron microscopy was performed to observe the morphology of exosomes (scale bar = 1 μm, arrows point to exosomes), and Western blot was used to analyze the expression of exosome surface marker protein

We identified the isolated BMSCs by detecting the pluripotent differentiation potential and surface markers of the cells. Microscopic observation showed that BMSCs formed calcium nodules after osteogenic differentiation induction, and the radioactive center was orange-red after staining with alizarin red. After BMSCs were induced to differentiate into adipogenesis, fine lipid droplets appeared in the cells (Fig. 1a). These data indicated that BMSC had both the ability of osteogenic and adipogenic differentiation. Flow cytometry results showed that BMSCs were immunopositive for markers of mesenchymal stromal stem cells namely CD90 and immunonegative for hematopoietic markers namely CD34 (Fig. 1b). BMSC-derived exosomes were identified by morphological observation and marker protein detection. By transmission electron microscopy, membranous vesicles of uniform size, round or oval shape, with clear margins and double lipid membranes surrounding them can be seen. Western blot results showed that the expression of exosome surface marker protein CD63 was significantly higher than BMSC lysate (Fig. 1c).

### Therapeutic effect of BMSC-derived exosomes on POF

**Fig. 2:**
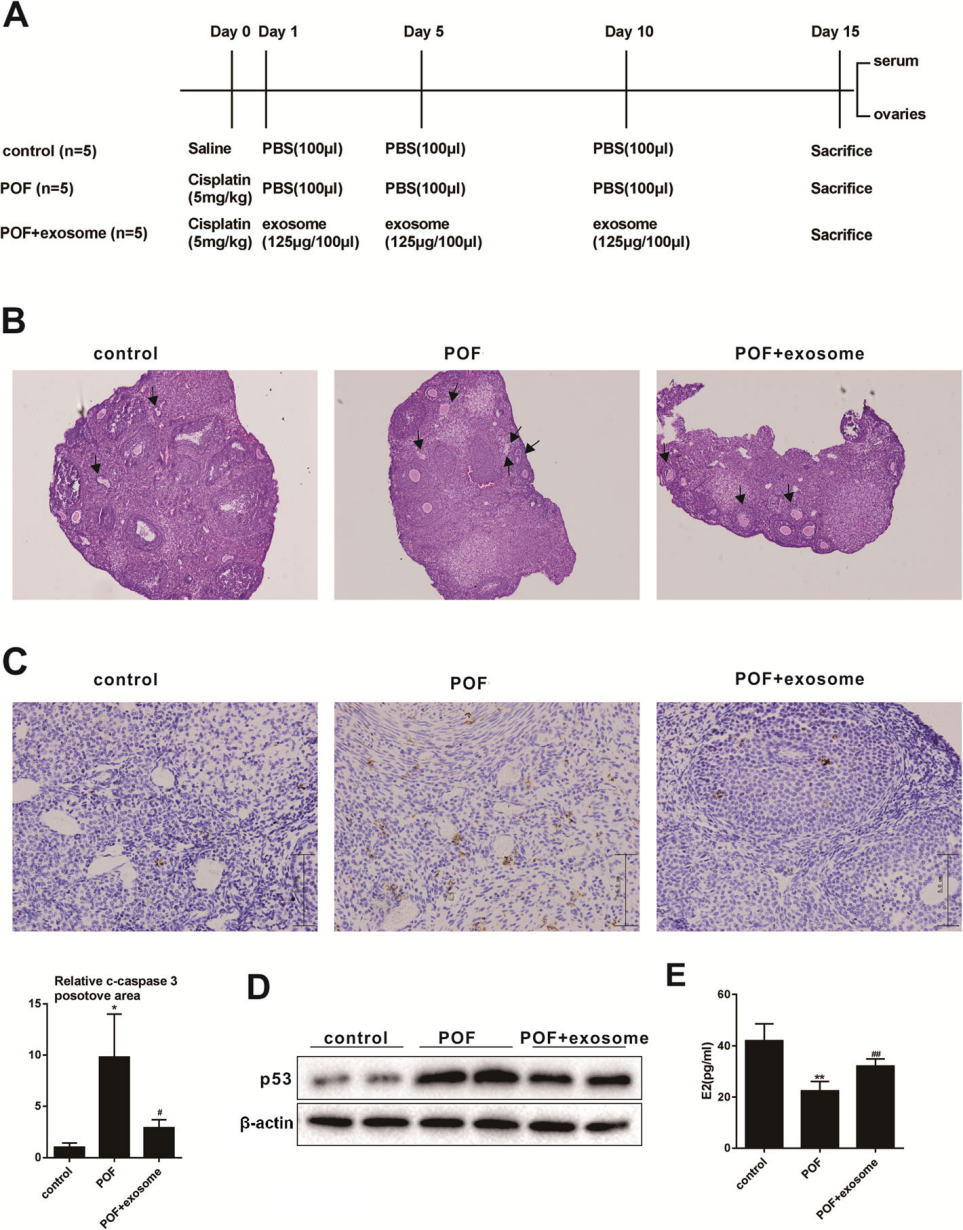
Therapeutic effect of BMSC-derived exosomes on POF. **a** Protocol for mouse model of POF. **b** HE staining of ovarian tissue was performed to observe the morphological differences of follicles (under (× 100) magnification, arrows point to atretic follicles). **c** The difference in the expression of cleaved-caspase3 in the ovarian tissue was analyzed by immunohistochemistry (scale bar, 5.0 μm) ^*^P<0.05 vs.control group, ^#^*P*<0.05 vs. POF group. **d** The difference expression of P53 in the ovarian tissue was detected by Western blot. **e** The concentration of E2 in the serum was detected by ELISA ^**^P<0.01 vs.control group, ^##^*P*<0.01vs. POF group. Data are expressed as mean ± standard deviation (SD)

The therapeutic effect of BMSC-derived exosomes on POF was evaluated by injecting BMSC-derived exosomes into a POF mouse model (protocols were shown in Fig. 2a). HE staining ovarian tissue experiment showed that the mice had large and abundant follicles, abundant follicular fluid, and multiple corpus luteum in the control group. The follicles in the POF group were few and mostly primitive or initial follicles, and the atresia follicles formed by granule cell damage increased, and the interstitial increased. Compared with the POF group, the atresia follicles in the POF + exosome group decreased, while the corpus luteum increased (Fig. 2b). Immunohistochemistry detection of cleaved caspase 3 was performed on the ovarian tissues. Compared with the control group, the expression of c-caspase3 was significantly up-regulated in the POF group, while this up-regulation of c-caspase3 was inhibited after exosome injection (Fig. 2c). Western blot was performed to detect the expression level of P53 in the ovarian tissue. Results showed that the expression of P53 in the POF group was increased compared with the control group, while the expression of P53 was dramatically lower in the POF + exosome group than that in the POF group (Fig. 2d). The concentration of E2 in the serum was analyzed by ELISA. It was found that the concentration of E2 in the POF group decreased compared with the control group, and the concentration of E2 in the POF + exosome group was increased relative to the POF group (Fig. 2e). From the above experimental results, it could be seen that BMSC-derived exosomes could improve POF.

### BMSC-derived exosomes protect granulosa cells from cisplatin damage by carrying microRNAs

**Fig. 3:**
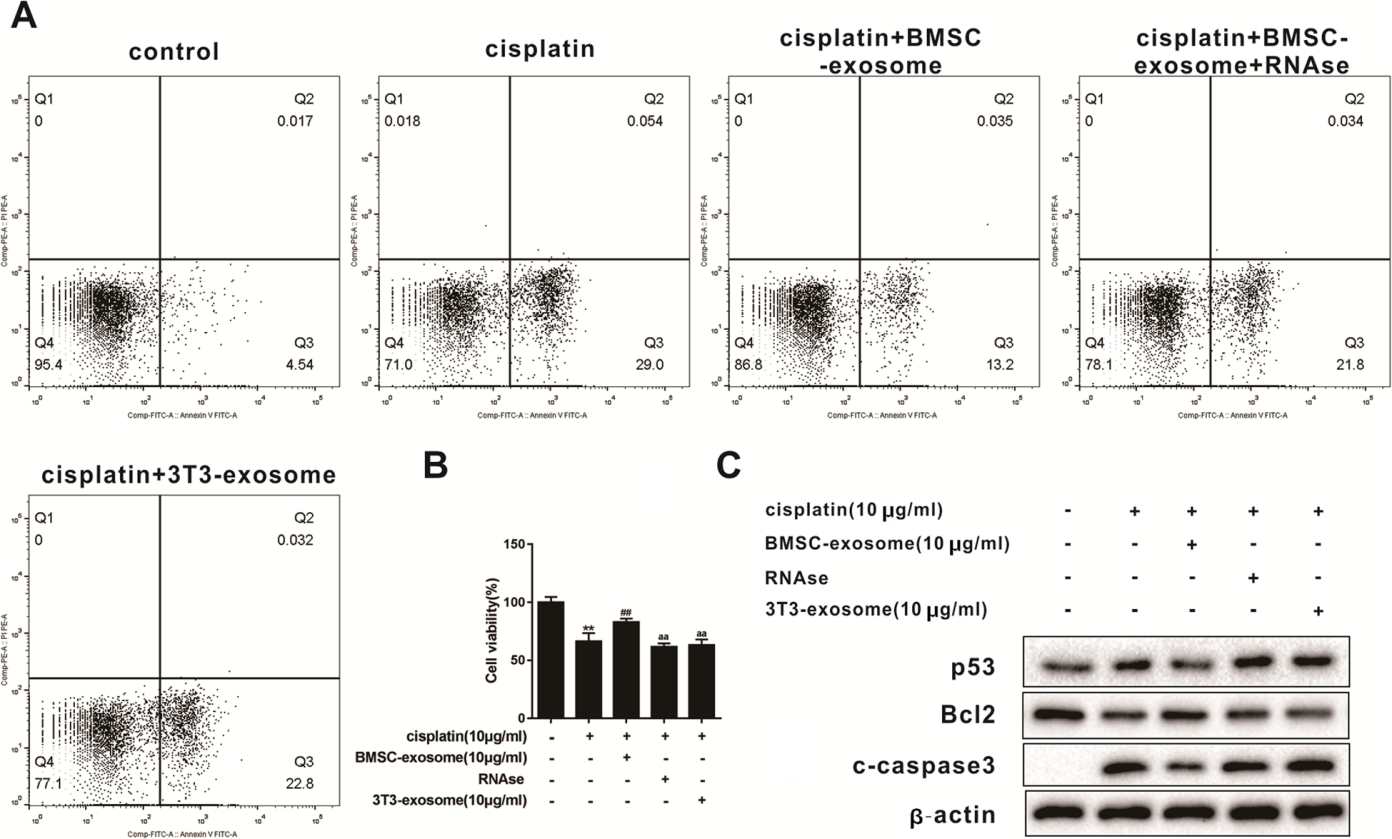
Protective effect of the RNA carried by BMSC-derived exosomes on granulosa cells damaged by cisplatin. **a** Analysis of the ratio of early apoptotic cells in five experimental groups by flow cytometry analysis. **b** Cell viability in the above five experimental groups were detected^**^P<0.01vs.control group, ^##^*P*<0.01vs.cisplatin group , ^aa^*P*<0.01 vs.cisplatin + BMSC-exosome group. **c** Western blot was used to detect differences in the expression of apoptosis-related proteins. Data are expressed as mean ± standard deviation (SD). Each experiment was repeated three times

Flow cytometry analysis showed that compared with the control group, the proportion of early apoptotic cells was increased in the cisplatin group, while this increase was reversed by BMSC-derived exosomes. This indicated that BMSC-derived exosomes inhibited the damage of granulose cells to cisplatin. When BMSC-derived exosomes were treated with RNase, the proportion of early apoptotic cells was increased compared with cisplatin + BMSC exosome group, indicating that the RNA in BMSC-derived exosomes inhibited granulose cells damage to cisplatin (Fig. 3a). The same therapeutic result was reached in the cell viability assay (Fig. 3b). Western blot was performed to detect the expression difference of apoptosis-related proteins. Results showed that compared with the cisplatin group, the expression of P53 protein and c-caspase3 protein was down-regulated and the expression of Bcl2 was up-regulated in the cisplatin + BMSC exosome group. When BMSC-derived exosomes were treated with RNase, the expression of P53 protein and c-caspase3 protein were up-regulated and the expression of Bcl2 was down-regulated compared to cisplatin + BMSC exosome group (Fig. 3c). These results consistently showed that the RNA in BMSC-derived exosomes had a strong protective effect on cisplatin-induced ovarian granulosa cell damage.

### miR-664-5p targets p53 to regulate apoptosis of granulosa cells

**Fig. 4:**
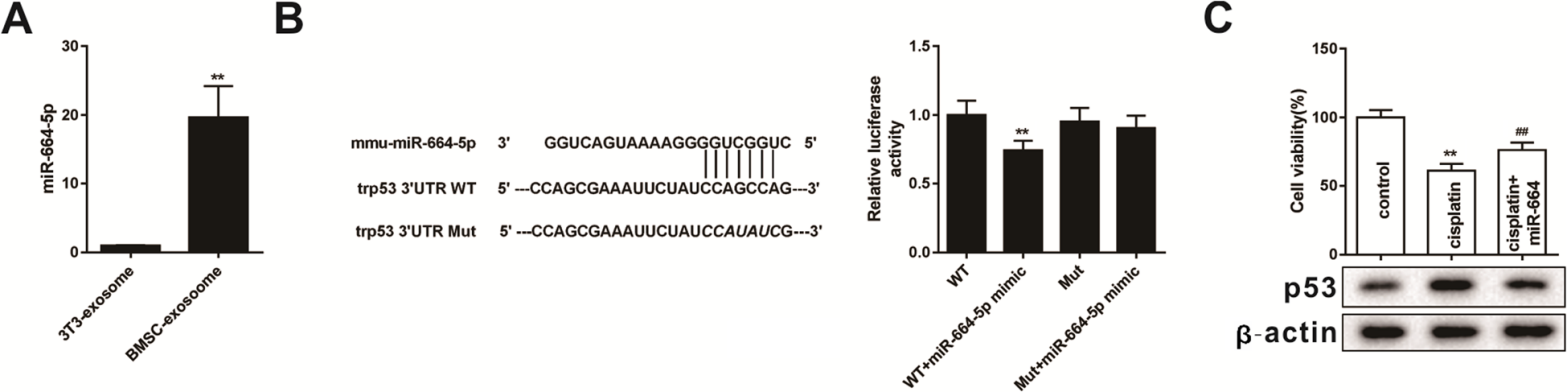
miR-664-5p targets p53 during apoptosis of granulosa cells. **a** qRT-PCR was used to detect the expression of miR-664-5p in exosomes of different cells^**^P<0.01vs.3T3-exosome group. **b** After transfection of miR-664-5p mimic and wild-type or mutant vectors into mouse ovarian granulosa cells, dual-luciferase reporter genes were performed to determine luciferase activity^**^P<0.01 vs. WT group. **c** Cell viability and the expression of p53 protein were measured after overexpression of miR-664-5p in mouse ovarian granulosa cells^**^P<0.01 vs.control group, ^##^*P*<0.01 vs. cisplatin group. Data are expressed as mean ± standard deviation (SD). Each experiment was repeated three times

Two databases, Target scan and microT-CDS, were used to search for the miRNA intersection predicted to target p53. A total of 21 miRNA intersections were found. We chose the top 10 miRNA from total miRNAs to verify their expression level by using qRT-PCR. The results indicated that miR-664-5p was highly expressed in BMSC-derived exosomes (Fig. 4a). Therefore, miR-664-5p was selected as the follow-up study object. From the results of dual-luciferase reporter assay, it was found that miR-664-5p bound to the 3′UTR of p53, which resulted in decreased relative luciferase activity in response to miR-664-5p mimic (Fig. 4b). This experimental result confirmed that miR-664-5p targeted p53 of cells. When miR-664-5p was overexpressed in mouse ovarian granulosa cells, cisplatin damage to granulosa cells was inhibited, and the expression of p53 protein was down-regulated (Fig. 4c). Briefly, we found that miR-664-5p targeted p53 of cells and inhibited granulosa cells damage to cisplatin.

### Protective effect of miR-664-5p carried by BMSC-derived exosomes on granulosa cells

**Fig. 5:**
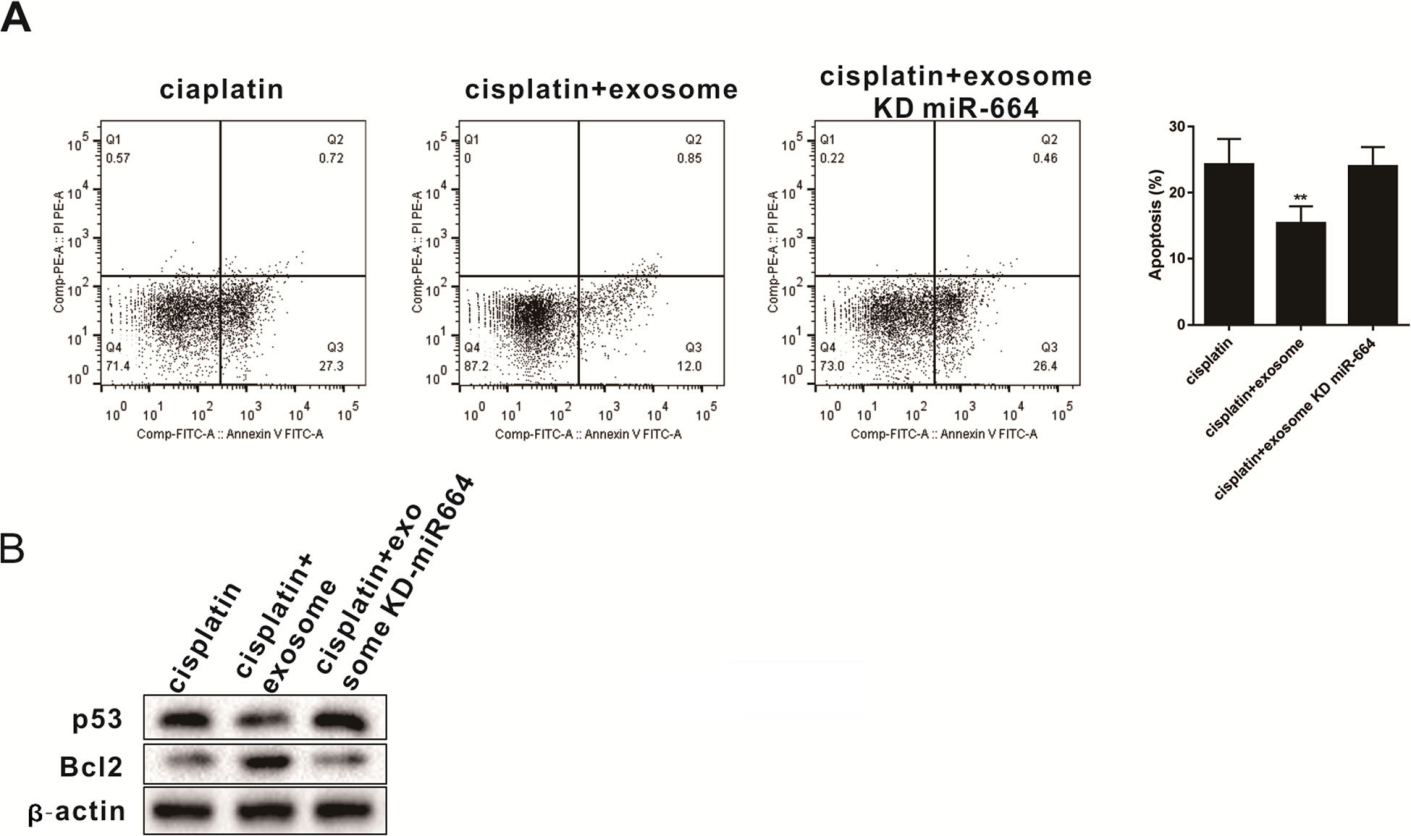
miR-664-5p carried by BMSC-derived exosomes protects granulosa cells. **a** Flow cytometry analysis was used to analyze granulosa cells apoptosis after reducing the expression of miR-664-5p in BMSC-derived exosomes^**^P<0.01vs. cisplatin group. **b** Western blot was performed to detect the expression of p53 and Bcl2 in granulosa cells after knocking out the miR-664-5p in BMSC. Data are expressed as mean ± standard deviation (SD). Each experiment was repeated three times

To further validate the protective role of miR-664-5p carried by exosome on granulosa cells, we isolated exosomes from BMSCs after miR-664-5p knockout and observed its effect on granulosa cell apoptosis. Results showed that the proportion of early apoptotic granulosa cells induced by cisplatin was increased, which reversed the protective effect of BMSC-derived exosomes on cells (Fig. 5a). Western blot was used to detect the expression of p53 and Bcl2 in granulosa cells. Knockout of miR-664-5p in BMSC reversed the inhibition of p53 and the promotion of Bcl2 expression in granulosa cells by BMSC-derived exosomes (Fig. 5b). The above experiments confirmed that miR-664-5p in BMSC-derived exosomes had a protective effect on granulosa cells.

## Discussion

Premature ovarian failure (POF) is defined as ovarian insufficiency before the age of 40 [16], and POF seriously affects women’s reproductive health. Current treatments include hormone therapy [17], oocyte and embryo cryopreservation and cryopreservation [18], ovarian tissue transplantation, and stem cell transplantation [19]. However, these methods have disadvantages such as poor therapeutic effect and poor prognosis. Therefore, there is an urgent need to provide new strategies for the clinical treatment of POF. This research focused on the protective mechanism of BMSC-derived exosomes on POF, and confirmed that BMSC-derived exosomes could improve POF, and revealed that miR-644-5p carried by BMSC-derived exosomes protected the function of ovarian granule cells by targeting p53 of cells.

Exosomes can mediate cellular communication through target cell internalization, ligand-receptor interaction, or lipid membrane fusion [20]. In recent years, more and more studies have confirmed that stem cell-derived exosomes are effective in treating diseases such as liver failure and heart failure by transferring protein and RNA cells [21, 22]. It has been reported that in the cisplatin-induced POF model, human umbilical cord mesenchymal stem cell (huMSC)-derived exosomes can significantly up-regulate the expression of the anti-apoptotic protein Bcl-2 and down-regulate the expression of the pro-apoptotic protein c-caspase-3, which indicates that huMSC-exosomes have a protective effect on cisplatin-induced apoptosis of ovarian granulosa cells [23]. In this study, the effect of BMSC-derived exosomes on POF was explored in a cisplatin-induced POF mouse model. It was found that BMSC-derived exosomes up-regulated the protein level of Bcl-2 and down-regulated the protein level of c-caspase-3 to protect granulosa cells from cisplatin injury. However, when exosomes were treated with RNase, the protective effect of BMSC-derived exosomes on cells was lost. This indicated that it was the RNA in BMSC-derived exosomes that had a protective function against cell damage. Therefore, we needed to solve a key issue in this study, which was to identify the miRNAs in BMSC-derived exosomes.

Exosomes contain many miRNAs that regulate the biological function of cells through intercellular shuttles. Many studies have reported that miRNAs carried by exosomes play a key role in stem cell-mediated tissue function repair [24, 25]. miR-21 in stem cells can improve chemotherapy-induced ovarian damage in rats by targeting PDCD4 and PTEN [26]. However, the miRNAs of BMSC-derived exosomes in granulosa cells remains largely unclear. Our studies indicated that miR-664-5p was overexpressed in BMSC-derived exosomes. Overexpression of miR-664-5p in granulosa cells could inhibit the damage of granulosa cells to cisplatin. Knockout of miR-664-5p in BMSC reversed the protective effect of BMSC-derived exosomes on granulosa cells. However, the mechanism by which miR-664-5p carried by BMSC-derived exosomes protects granulosa cells has not been fully elucidated. It has been reported that p53 is one of the tumor suppressor proteins that induces tumor cell cycle arrest and regulates programmed apoptosis in cancer cells [27]. Studies have found that Amarogentin can promote the apoptosis of liver cancer cells by up-regulating p53 of cells [28]. Another research points out that the expression of p53 is abnormally up-regulated in human colon cancer cells, and the activation of p53 can induce apoptosis of cancer cells and reduce cell migration [29]. In addition, studies have shown that the expression of p53 is significantly increased in doxorubicin-induced ovarian granulosa cells, and the expression of apoptosis-related proteins is also increased [30]. However, the role of p53 in granulosa cell apoptosis has not been fully elucidated. In the present study, we found that the expression of p53 was increased in ovarian granulosa cells induced by cisplatin. Further experiments showed that p53 was involved in the regulation of granulosa cell apoptosis by miR-664-5p.

In conclusion, our results showed that BMSC-derived exosomes played a key role in restoring ovarian function in a cisplatin-induced POF mouse model, mainly by delivering miR-644-5p to granulosa cells to regulate p53 expression of cells and thereby inhibited ovarian granules apoptosis. Our current study clarified the potential molecular mechanisms of BMSC-derived exosome-mediated ovarian function recovery and provided new strategies and directions for the treatment of POF.

## Data Availability

Not applicable.

## Abbreviations

BMSC: Bone mesenchymal stem cell
DLR: Dual-Luciferase Reporter
ELISA: Enzyme linked immunosorbent assay
huMSC: human umbilical cord mesenchymal stem cell
miRNAs: microRNAs
POF: Premature ovarian failure
PI: propidium iodide
qRT-PCR: Quantitative Real-time PCR
SD: standard deviation
